# Overcoming the challenges of studying conservation physiology in large whales: a review of available methods

**DOI:** 10.1093/conphys/cot006

**Published:** 2013-05-15

**Authors:** Kathleen E. Hunt, Michael J. Moore, Rosalind M. Rolland, Nicholas M. Kellar, Ailsa J. Hall, Joanna Kershaw, Stephen A. Raverty, Cristina E. Davis, Laura C. Yeates, Deborah A. Fauquier, Teresa K. Rowles, Scott D. Kraus

**Affiliations:** 1 John H. Prescott Marine Laboratory, Research Department, New England Aquarium, Boston, MA 02110, USA; 2 Biology Department, Woods Hole Oceanographic Insitution, Woods Hole, MA 02543, USA; 3 Southwest Fisheries Science Center, National Marine Fisheries Service, National Oceanic and Atmospheric Administration, La Jolla, CA 92037, USA; 4 Sea Mammal Research Unit, Scottish Oceans Institute, St Andrews KY16 8LB, UK; 5 Animal Health Centre, Abbotsford, BC, Canada V6M 1A2; 6 Mechanical and Aerospace Engineering, University of California, Davis, CA 95616, USA; 7 National Marine Mammal Foundation, San Diego, CA 92106, USA; 8 Marine Mammal Health and Stranding Response Program, National Marine Fisheries Service, National Oceanic and Atmospheric Administration, Silver Spring, MD 20910, USA; 9 John H. Prescott Marine Laboratory, Research Department, New England Aquarium, Boston, MA 02110, USA;

**Keywords:** Blow, biopsy dart, Cetacea, faecal samples, non-invasive, visual health assessment

## Abstract

Large whales are subjected to a variety of conservation pressures that could be better monitored and managed if physiological information could be gathered readily from free-swimming whales. However, traditional approaches to studying physiology have been impractical for large whales, because there is no routine method for capture of the largest species and there is presently no practical method of obtaining blood samples from free-swimming whales. We review the currently available techniques for gathering physiological information on large whales using a variety of non-lethal and minimally invasive (or non-invasive) sample matrices. We focus on methods that should produce information relevant to conservation physiology, e.g. measures relevant to stress physiology, reproductive status, nutritional status, immune response, health, and disease. The following four types of samples are discussed: faecal samples, respiratory samples (‘blow’), skin/blubber samples, and photographs. Faecal samples have historically been used for diet analysis but increasingly are also used for hormonal analyses, as well as for assessment of exposure to toxins, pollutants, and parasites. Blow samples contain many hormones as well as respiratory microbes, a diverse array of metabolites, and a variety of immune-related substances. Biopsy dart samples are widely used for genetic, contaminant, and fatty-acid analyses and are now being used for endocrine studies along with proteomic and transcriptomic approaches. Photographic analyses have benefited from recently developed quantitative techniques allowing assessment of skin condition, ectoparasite load, and nutritional status, along with wounds and scars from ship strikes and fishing gear entanglement. Field application of these techniques has the potential to improve our understanding of the physiology of large whales greatly, better enabling assessment of the relative impacts of many anthropogenic and ecological pressures.

## Abstract (precis)

A description and comparison of the four major methods available for studying conservation physiology of large whales, namely analysis of faecal, respiratory vapour, and skin/blubber biopsy samples, and photographs.

Large whales experience numerous anthropogenic pressures that warrant closer study from a conservation physiology perspective. Most large cetacean species were subjected to severe overexploitation by commercial whaling over the last two centuries (Clapham *et al.*, 1999). Since the international moratorium on commercial whaling in 1986, some populations have recovered to varying degrees, but most have not yet reattained their historical numbers, and most populations are still threatened or endangered (Kraus *et al.*, 2005; Freeman, 2008; Lotze and Worm, 2008; Reynolds *et al.*, 2009; IUCN, 2012; McClenachan *et al.*, 2012). Large whale populations today are facing a novel mix of anthropogenic impacts, including ship strikes, entanglement in fishing gear, exposure to anthropogenic noise (e.g. shipping noise, military sonar, and seismic exploration), chronic exposure to a variety of toxins and pollutants, and possible impacts of global climate change that include potential shifts in prey availability as well as changes in ice cover and human use of polar oceans (Doney *et al.*, 2011; Pompa *et al.*, 2011; Davidson *et al.*, 2012).

Many of these conservation pressures are thought to elicit acute or chronic physiological responses that may be detectable in individual animals before population-level impacts on health, mortality, or fecundity become apparent (Wikelski and Cooke, 2008; Cooke and O'Connor, 2010). Examples of these responses include elevations in stress-associated hormones, declines in reproductive hormones, depressed immune responses, and declines in body condition (Wikelski and Cooke, 2008; Cooke and O'Connor, 2010; Homyack, 2010). Such physiological changes could be used as indicators to monitor the occurrence, extent, severity, and cumulative effects of various conservation-relevant pressures on large whales. Physiological measures can also help to elucidate specific causes and changes in patterns of mortality and reproduction (Wikelski and Cooke, 2008; Cooke and O'Connor, 2010). A recent workshop of the United States National Marine Fisheries Service (Marine Mammal Breath Workshop, La Jolla, CA, USA, 13–14 August 2012, a result of which is this review) concluded that physiological measures could be useful for monitoring, studying, and managing various conservation crises that periodically affect large whale populations, potentially including long-term impacts of oil spills, unusual mortality events, episodes of unexplained reductions in reproduction, and monitoring of individual whales to assess the need for possible management intervention in unusual situations (e.g. an entangled whale or a whale that has swum up a freshwater river). Such measures could also be informative for assessing the effects of chronic sources of disturbance (shipping noise, seismic exploration, and climate change). Furthermore, cetaceans have been proposed as model species to use as ‘ecosystem sentinels’, particularly migratory mysticetes and polar species, due to their use of multiple marine ecosystems across large geographical areas (Moore, 2008; Fossi *et al.*, 2012). There is therefore increasing interest in development and application of conservation physiology techniques for large whales.

Unfortunately, large whales are perhaps the most difficult taxon of vertebrates to study physiologically. Perhaps the greatest logistical difficulty is that there is currently no routine method of capturing and restraining a live large whale unharmed. This means that there is no practical means of obtaining that most classic of physiological samples, a blood sample, without killing the animal. Furthermore, there are no facilities that can accommodate species larger than ∼8 m in body length (e.g. larger than killer whales, *Orcinus orca*), which limits application of many classic physiological methods to whales. This is particularly a problem for research on basic physiology of baleen whales, because no mysticete species are routinely kept in captivity. Compounding the problem, cetaceans in general are difficult to observe and spend only brief periods of time at the surface. The result is that our present understanding of large whale physiology is limited compared with most other vertebrate taxa.

Fortunately, several techniques have recently become available that allow direct study of many physiological parameters through a variety of non-lethal, minimally invasive approaches. We review the major methods currently available for non-lethal assessment of cetacean physiology, focusing on techniques that can be applied to faecal samples, respiratory vapour samples, blubber and skin biopsy samples, and photographic data. Our purpose is to encourage conservation physiologists to further develop and apply these techniques to the conservation and management of large whales.

## Faecal samples

Faecal samples have long been studied for information specific to digestive physiology, particularly intestinal parasitology and diet analysis, but more recently mammalian faeces have been shown to contain very high concentrations of steroid hormones as well as several other measures of interest (Wasser *et al.*, 1988; Schwarzenberger *et al.*, 1996a; Brown and Wildt, 1997; Palme, 2005). As a result, there has been increasing interest in applying these faecal analytical techniques to large whales.

### Faecal sample collection from large whales

**Figure 1:**
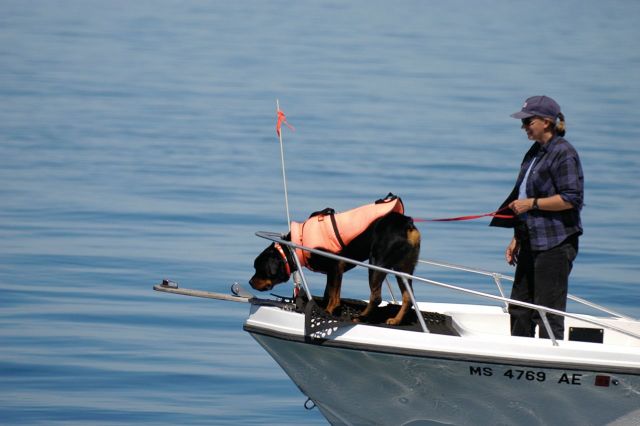
faecal samples from large whales can be located opportunistically, via focal follows, or with trained dogs. This photograph illustrates use of a trained scenting dog for collection of North Atlantic right whale (*Eubalaena glacialis*) faecal samples. Once scent is detected, the boat is steered into the wind until the sample is located, upon which the dog receives a tennis-ball reward (Rolland *et al.*, 2007). (Photo: New England Aquarium, Fisheries and Oceans Canada permit #MAR-SA-2005-2007.)

Faecal samples have been collected successfully from multiple species of large whales, including North Atlantic right whales (NARWs; *Eubalaena glacialis*; Weisbrod *et al.*, 2000; Rolland *et al.*, 2005, 2007), sperm whales (*Physeter macrocephalus*; Smith and Whitehead, 2000), killer whales (Ayres *et al.*, 2012), humpback whales (*Megaptera novaeangliae*; Roman and McCarthy, 2012), blue whales (*Balaenoptera musculus*; Lefebvre *et al.*, 2002), and Blainville's beaked whales (*Mesoplodon densirostris*; D. Claridge, personal communication, Bahamas Marine Mammal Research Organization, Abaco, Bahamas). Many species defaecate at the surface prior to diving and, in the case of right whales, also in the vicinity of surface active (courtship) groups. Faecal samples have been located opportunistically during whale population surveys, and can also be collected by dedicated focal following of individuals or groups of whales. In the case of Blainville's beaked whales, the animals defaecate while travelling ∼3–4 m subsurface during intervals between deep dives, and samples have been collected successfully by towing a researcher in snorkel gear who follows the whales underwater and dives to collect samples using a small dipnet and Ziploc bag (D. Claridge, personal communication, Bahamas Marine Mammal Research Organization, Abaco, Bahamas). Scent-detection dogs working from the bow of a boat have been successfully used to locate floating faecal samples of NARWs (Rolland *et al.*, 2006) and killer whales (Ayres *et al.*, 2012). In right whales, use of detection dogs increased sampling rates over 4-fold, with dogs locating samples from as far away as a nautical mile (Rolland *et al.*, 2006; Fig. 1).

Faecal sample consistency in large whales varies with species (and sometimes season and diet), from well-formed floating semi-solid clumps (e.g. right whales and sperm whales) to a more fluid, dispersed plume (e.g. humpback whales). Floating semi-solid samples can be scooped from the water surface using a fine-mesh nylon dipnet, draining off as much seawater as possible. More fluid faeces can be scooped into a plastic container or bag, although this can result in a sample containing a high ratio of saltwater to faeces. In the latter case, the possibility of migration of polar hormone metabolites into the seawater should be examined.

## Faecal hormone analysis

Many recent faecal studies in large whales have focused on faecal steroid hormones (e.g. Ayres *et al.*, 2012; Rolland *et al.*, 2012). In vertebrates, steroid hormones (androgens, oestrogens, progestagens, mineralocorticoids, and glucocorticoids), are primarily cleared from blood by the liver and excreted via two routes: (i) secretion in bile to the gut lumen, from which the hormones are voided in faeces; and (ii) conjugation to polar side-groups and eventual voiding via the urine (Palme *et al.*, 1996; Möstl and Palme, 2002; Busch and Hayward, 2009; Sheriff *et al.*, 2010; Wasser *et al.*, 2010). In faeces, steroid hormones accumulate over ∼4–48 h, depending on the species-specific gut transit time (e.g. Wasser *et al.*, 2010). Thus, faecal hormone measures are usually interpreted to reflect average endocrine activity of the previous day or two, unlike a blood sample that represents a single point in time. Upon excretion, the final concentration of steroids per gram of faeces is often several orders of magnitude higher than it is per millilitre of plasma (e.g. Wasser *et al.*, 1988), such that even a small faecal sample typically contains sufficient hormone for multiple analyses.

Steroid hormones are usually altered chemically during gut transit, with each parent hormone producing a family of related ‘faecal hormone metabolites’ (FHMs; Brown and Wildt, 1997; Palme *et al.*, 2005). The chemical identity of these FHMs is species specific, and in most mammalian taxa (including cetaceans) is currently unknown. Thus, techniques that recognize only certain specific steroids, such as high-performance liquid chromatography (HPLC) and mass spectrometry, are typically not used for FHM analysis. Rather, antibody-based techniques, such as immunoassays, are the preferred method, because many antibodies can detect not only the parent hormones but also certain of the FHMs. In the last two decades, several antibodies have been identified that recognize common mammalian FHMs across a wide variety of taxa (Schwarzenberger *et al.*, 1996b; Wasser *et al.*, 2000, 2010; Graham *et al.*, 2001).

Owing to the variety of FHMs that may be present, every assay must be validated for each new species studied. Essential validations include parallelism tests, which verify that the antibody binds well to FHMs of that species, and accuracy tests (also known as ‘matrix tests’), which determine whether the assay can distinguish low from high hormone concentrations in the presence of faecal matrix (Grotjan and Keel, 1996). A second set of validations, often termed ‘physiological validations’, must then be performed to test whether the measured FHM concentrations truly reflect the physiological state of the animal (Palme, 2005). In large whales, physiological validation can be accomplished via study of animals of known physiological state (e.g. adult males, pregnant females, lactating females, etc.) to test whether FHM measures correctly assign animals to their (independently known) physiological state. Ideally, normal ranges for each FHM should be established for each demographic group under study (age, sex, and reproductive state) and sometimes for different seasons and diets as well (Touma and Palme, 1996; Goymann, 2012). It is particularly important to delineate the normal range in a healthy control population. For example, during long-term chronic stress it is possible for glucocorticoid levels to fall below normal values (Cyr and Romano, 2009). Such a drop below normal may only be recognized and interpreted correctly if the normal range in a healthy, relatively unstressed, control population has been adequately described or, alternatively, if the same population is available for study before vs. after exposure to a given stressor.

A host of studies in terrestrial mammals have demonstrated that if the aforementioned validations are carefully performed, FHMs accurately reflect reproductive state, adrenal activity, and even thyroid activity, and can be useful as non-invasive measures of reproduction, stress, nutritional status, and metabolic rate (Schwarzenberger *et al.*, 1996b; Wasser *et al.*, 2000, 2010; Schwarzenberger, 2007; Busch and Hayward, 2009; Sheriff *et al.*, 2010). The initial validations are commonly performed in small, well-studied populations with known individuals, but once validations are successful the technique can frequently be applied to larger, less well-studied populations. The same approach has been used successfuly in faecal hormone studies of terrestrial species, many of which began with validations performed on known individuals, with later extension of the techniques to larger, less well-known populations (e.g. Hunt and Wasser, 2003; followed by Wasser *et al.*, 2004)*.*

In large whales, faecal hormone techniques have been applied successfully to both mysticetes and odontocetes, with the most thorough studies so far focusing on right whales and killer whales. Over a decade of research on western NARWs has shown that it is possible to collect large numbers of faecal samples from free-swimming whales for non-invasive studies of reproductive and stress endocrinology (Rolland *et al.*, 2005, 2006, 2012; Hunt *et al.*, 2006). Analysis of reproductive hormone metabolites (oestrogens, progestins, and androgens), in combination with life history data from photographically identified right whales, has demonstrated that levels of faecal hormones discriminate accurately between right whales of known reproductive state (Rolland *et al.*, 2005). The ratio of faecal androgens to oestrogens is 100% accurate for determining the gender of samples from right whales of known sex. Mature males have more than double the faecal androgen level of younger males, highly elevated levels of faecal progestins correctly predict pregnancy, and lactating females can be distinguished from non-lactating, non-pregnant females via higher faecal oestrogen and androgen levels.

In addition, faecal glucocorticoid (GC) analysis has been validated for evaluation of relative levels of adrenocortical activity and physiological stress in right whales (Hunt *et al.*, 2006). Baseline levels of faecal GCs varied with age and reproductive state and, therefore, GCs must be interpreted with knowledge of the age-class and reproductive condition of the whale (Hunt *et al.*, 2006); fortunately, this information can often be gathered via the other steroid hormones. Highly elevated GCs in whales in a known stressed condition (i.e. severe entanglement in fishing gear), compared with expected GC levels for that whale's demographic class, have provided biological validation that glucocorticoid FHMs accurately reflect adrenocortical activation and physiological stress (Hunt *et al.*, 2006). Finally, measurement of faecal GCs in tandem with acoustic studies has provided the first evidence that exposure to low-frequency underwater noise from ships may be associated with chronic stress in right whales (Rolland *et al.*, 2012).

Similar techniques have recently been applied to odontocetes as well, notably the southern resident killer whales in Puget Sound (Ayres *et al.*, 2012). This population has declined in recent years, and theories for the decline include nutritional stress from depleted stocks of prey (chinook salmon) and/or seasonal disturbance related to high levels of vessel traffic in close proximity to the whales. By using a combination of faecal GCs (interpreted as a measure of both types of stressors) and faecal thyroid hormone metabolites (interpreted as a measure of nutritional stress specifically), Ayres *et al.* (2012) were able to assess the relative impacts of the two types of stressors. The authors concluded that the more important factor impacting the population is nutritional stress.

### Non-endocrine faecal measures

Faecal samples have long been used for diet analysis, traditionally via visual inspection of skeletal elements and more recently using genetic methods and stable isotope analysis (e.g. killer whale, Hanson *et al.*, 2010). Digestive efficiency can also be assessed via comparison of faeces to the composition of prey. Using this approach, Swaim *et al.* (2009) demonstrated that while the NARW's primary prey contains a high proportion of wax esters relative to other lipids, NARW faeces contain very few wax esters. This indicates that NARWs are highly efficient at digestion of wax esters—an unusual property among mammals. Such approaches could be useful for modelling of habitat quality, e.g. determining the prey base necessary to support a given population size in a given habitat.

Faecal samples can also be used to monitor exposure to toxins and parasites. The algal toxin domoic acid, which has caused dramatic mortality events in pinnipeds, has been detected in humpback and blue whale faeces, particularly during episodes of toxic algal blooms (Lefebvre *et al.*, 2002). During a 6 year study of NARWs, more than one-quarter of faecal samples indicated annual exposure to domoic acid and 70–80% to paralytic shellfish toxins, with 22% of samples showing concurrent exposure to both neurotoxins (Doucette *et al.*, 2012). The protozoan parasites *Cryptosporidium* spp. and *Giardia* spp. have been detected in both NARW faeces and bowhead whale (*Balaena mysticetus*) faeces (Hughes-Hanks *et al.*, 2005). Prevalence of the two parasites was much higher in NARWs (71% of NARWs were exposed to *Giardia*, and nearly a quarter to *Cryptosporidium*), but genotyping of the isolates is needed to determine the species and potential source of these organisms.

Finally, faecal matter also includes DNA from a variety of sources, including the host animal, the prey species, and gut microflora (Valentini *et al.*, 2009). Characterization of the gut microbiome of large whales via genomic analyses may be useful for monitoring digestive physiology and disease. In mammals, the composition of gut microflora often shows dramatic shifts associated with stress and disease (e.g. Guarner and Malagelada, 2003; Bailey *et al.*, 2010). Additionally, recent studies in several species indicate that the composition of the gut microflora plays an important role in host energy metabolism (Cani and Delzenne, 2007), stress physiology (Dinan and Cryan, 2012), and immune response (Round and Mazmanian, 2009).

The host's own DNA is also present in faeces. Host DNA can be useful for identifying the individual whale (e.g. in cases where the sample was found floating among a group of whales), for associating analytical results with a particular individual, and for confirming the species. Generally, mammalian faecal DNA is highly degraded and presents some analytical challenges, including poor amplification success, ‘allelic dropout’ (heterozygotes appearing as homozygotes), and short fragment length. However, with selection of appropriate markers and modification of extraction techniques to eliminate faecal PCR inhibitors, genetic techniques have been successfully adapted to faeces (Pages *et al.*, 2009). In NARWs, approximately half of tested faecal samples could be successfully genotyped to the individual (Gillett *et al.*, 2010).

### Faecal sample analysis: advantages, disadvantages, and next steps

Faecal analysis offers a window into aspects of digestive, reproductive, adrenal, and thyroid physiology that have until now been difficult to study in large whales (Table 1). Faecal hormone analyses, in particular, have already proved useful for applied conservation questions in two endangered populations, i.e. elucidating effects of chronic noise in NARWs (Rolland *et al.*, 2012), and disentangling relative impacts of boat disturbance and food availability in southern resident killer whales (Ayres *et al.*, 2012). Faecal DNA and toxin analyses have recently been successful as well (see above).

**Table 1:** comparison of techniques currently available for study of conservation physiology of large whales

Sample type	Typical collection methods	Typical sampling rate	Positive aspects	Potential limitations	Information relevant to conservation physiology
Faeces	Locate visually or with dog	Low without dog	Non-invasive	Low sampling rate	Diet analysis
	Surface collection with scoop or net; subsurface collection with divers	Medium with dog	Extremely high steroid content (easily detectable)	Targeted sampling difficult	Endoparasites
			Well-established steroid hormone techniques	Individual not always known (cannot always be genotyped due to DNA degradation)	Lipophilic hormones
			Long ‘sampling time frame’ may enable study of chronic stress	Cannot sample fasting seasons	Fatty acid and stable isotope analysis of diet
			Repeated sampling possible		Toxin exposure (e.g. domoic acid)
					Gut microbiome and relationships to stress, immunity, and disease
					Some immunoglobulins and other hormones may be detectable (?)
Respiratory vapour (‘blow’)	Pole-based samplers	Medium	Non-invasive	Novel technique; many validations remain to be done	Several hormones detectable
	Remote-controlled devices possible (?)		Targeted biomarker sampling possible		May contain large variety of other detectable compounds (?)
	Different methods for droplets, exhaled breath condensate, and gases		Repeated sampling possible	Target biomarkers at trace concentratons	May be proxy for blood, as has been observed in human studies
			Wide range of metabolites can be studied simultaneously	Advanced detection strategies needed for quantitative analysis	Respiratory microbiome
					Host immune response
Epithelium and blubber biopsies	Biopsy dart used with crossbow, pole, or pneumatic rifle	Medium/high	Good sampling rate	Invasive; causes small wound	Lipophilic hormones in blubber
	Sloughed skin may also be collectable (?)		Many archived samples available	Permit restrictions	Lipid/fatty acid analysis: contaminent load (POPs), diet, age, etc.
			Tissue sample obtained; living cells present; high protein and nucleic acid content	Repeated sampling not always possible	Epidermal microbiome, skin lesions, and epidermal diseases
				‘Lag’ time of blubber hormones unknown	Epidemal proteomics (CYP450-related enzymes for contaminants, SRPs for stress studies)
			If sloughed, then non-invasive		Transcriptomic and genomic approaches possible (?)
Photographic analysis	Lateral view with boat-based photography	Very high	Non-invasive	External appearance only	Blubber reserves/nutritional state
			Best sampling rate		Epidermal lesions
	Dorsal view/body outline with aeroplanes or remote-control devices		Repeated sampling possible	Aeroplane-based photography has cost/safety issues	Ectoparasites
					Entanglement and injury
	Infrared thermography				Thermal physiology (infrared)

Novel, untested possibilities are indicated with question marks. POP = persistent organic pollutants; CYP450 = cytochrome P450; SRP = stress-response proteins.

Faecal sampling has several advantages; it is entirely non-invasive (i.e. the animal is not contacted during sampling), and FHMs tend to be present in very high concentrations and are readily detectable with cost-effective techniques. The development of faecal hormone analytical techniques for whales has benefited from a vast literature on terrestrial species. Finally, given that FHMs reflect a relatively long time frame of circulating hormone (often 1–2 days), FHMs may be well suited for distinguishing effects of acute vs. chronic environmental stressors (Busch and Hayward, 2009).

The major disadvantages to faecal sampling include low sampling rate, difficulty of targeted sampling (that is, focal whales may not defaecate while under observation), occasional uncertainty as to which individual the sample is from, and inability to collect samples during fasting seasons for some species, which precludes study of a potentially important part of the annual cycle (Table 1). For FHM analysis, each new species studied requires assay validations, physiological validations on known-state individuals, and establishment of normal ranges for different demographic groups. Other issues that have not been fully resolved include potential effects of diet and season, individual variation, possible degradation of FHMs after sample collection, and potential loss of polar hormone metabolites to seawater.

There are many additional novel faecal measures that should be investigated in large whales. Faecal thyroid hormones are a recent addition to the available endocrine panel (Wasser *et al.*, 2010; Ayres *et al.*, 2012), and faecal aldosterone offers the potential of additional nuanced information on adrenal activity. The potential exists to develop faecal immune measures, particularly immunoglobulin A, which in other mammals tends to increase in faeces during episodes of inflammation (e.g. human infants, Saarinen *et al.*, 2002) and other forms of stress (e.g. social stress in mice, Bundgaard *et al.*, 2012). Other possible measures of interest from faeces include various different oestrogens (estriol, estrone, etc.), reverse-triiodothyronine, and further development of genetic and genomic techniques.

## Respiratory samples (‘blow’)

Large whales produce clouds of ‘blow’ (exhaled droplets of condensed respiratory vapour) as they exhale at the surface. Individual whales usually blow several times during a single surfacing interval, and often they can be approached closely by boats during this time. The potential to sample part of this ‘blow cloud’ has not gone unnoticed, and in recent years it has been realized that blow may represent a valuable, entirely non-invasive, physiological sample that could be collected with relative ease. Recent developments in human breath research (Schubert *et al.*, 2004; Dummer *et al.*, 2011; Dweik, 2011) have accelerated interest in developing this novel method for cetaceans.

Human breath studies have shown that mammalian breath contains a mixture of volatile compounds in the gaseous phase (Miekisch *et al.*, 2004), as well as non-volatile compounds that occur in small aerosolized droplets (Kharitonov and Barnes, 2002; Grob *et al.*, 2008). Thousands of putative compounds have been identified in human breath (Bean *et al.*, 2012). Some of these have been shown to be physiologically relevant indicators of health and disease, such as breath biomarkers indicative of specific bacterial, fungal, and viral respiratory infections (Phillips *et al.*, 2010a, b, 2012; Chambers *et al.*, 2012), and others associated with various types of cancer (Miekisch *et al.*, 2004; Machado *et al.*, 2005; Mazzone, 2008; Horvath *et al.*, 2009; Fuchs *et al.*, 2010; Amann *et al.*, 2011). Breath analysis can also detect the presence of ingested compounds (Beck *et al.*, 2012), exposure to specific exogenous substances from the environment (Maniscalco *et al.*, 2006; Pleil, 2008), and changes in biomarkers that precede immunological events (Phillips *et al.*, 2004a, b) and stressful events (Turner *et al.*, 2013). The potential application of these measures to cetacean conservation physiology studies is obvious.

Human breath studies have shown that the sampling method can have profound effects on the analytical results (Bojko *et al.*, 2011). In humans, inert Tedlar^®^ bags are often used for collection of whole breath (Groves and Zellers, 1996; Steeghs *et al.*, 2007; Beauchamp *et al.*, 2008), while non-volatile components are often collected via cooling of exhaled breath to produce ‘exhaled breath condensate’ (EBC; Soyer *et al.*, 2006; Davidsson and Schmekel, 2010), and volatile compounds can be collected with vacuum canisters (Lindstrom and Pleil, 2002) or sorbent traps (Phillips, 1997; Ligor *et al.*, 2008; Mieth *et al.*, 2009; Bojko *et al.*, 2011). Analytical methods for human breath analysis have primarily relied on various forms of traditional mass spectrometry, but other alternative methods are also being developed, including ion mobility spectrometry (Baumbach, 2009), differential mobility spectrometry (Krebs *et al.*, 2005; Molina *et al.*, 2008), selected ion flow tube mass spectrometry (Diskin *et al.*, 2003; Smith and Spanel, 2011a, b; Spanel and Smith, 2011), colorimetric sensors that indicate the presence of fingerprints of breath compounds of interest (Mazzone *et al.*, 2007), and single compound detectors (e.g. nitrogen oxide sensors, Mondal *et al.*, 2011).

### Respiratory sample collection from large whales

**Figure 2:**
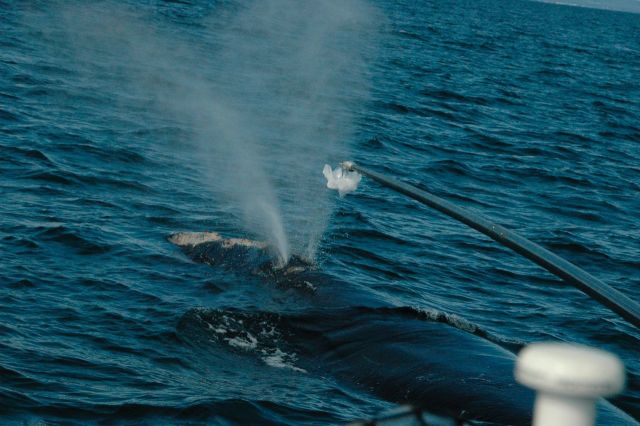
respiratory vapour samples (‘blow’) from large whales can be collected by a variety of pole-based or remote-controlled helicopter-based methods. This photograph shows collection of respiratory vapour (blow) droplets from a North Atlantic right whale (*Eubalaena glacialis*) using a nylon-fabric sampler suspended on the end of a carbon-fibre pole. (Photo: Amy Knowlton, New England Aquarium, SARA permit #325863, NMFS permit #14233.)

Several of the human breath-sampling methods described above have been successfully modified for cetaceans. Blow samples have been collected from small trained odontocetes in captivity by holding an inverted tube or other device directly over the animal's blowhole (Frere *et al.*, 2010; Thompson *et al.*, 2013). Blow droplets have been collected from both humpbacks and NARWs using a variety of sampling devices attached to long poles positioned over the blowholes. Pole-based samplers have included the following: nylon fabric suspended across a 15 cm ring or a plastic framework (NARW and humpback; Hogg *et al.*, 2009; Hunt *et al.*, 2012; Fig. 2), an inverted funnel (used with a stranded grey whale, *Eschrichtius robustus*; C. Davis, unpublished data), and Petri dishes (killer whales, Schroeder *et al.*, 2009). A remote-controlled helicopter has been used to collect blow droplets on Petri dishes attached to the helicopter skids (humpback whales, Acevedo-Whitehouse *et al.*, 2010). Recently, a remote-controlled ‘hexacopter’—a small, stable remote-controlled helicopter with six rotors—has been developed that shows potential for blow collection (W. Perryman, personal communication, NOAA SW Fisheries Science Center, La Jolla, CA, USA). Sample volume and sampling rate have generally been acceptable with the aforementioned techniques. These studies demonstrate that routine blow droplet collection from large whales is feasible. Finally, the potential exists to develop a device that could capture a portion of the exhaled air for analysis of gaseous components.

### Blow hormones

In 2009, detectable testosterone and progesterone were reported in blow droplets collected from both humpbacks and NARWs, as assessed with liquid chromatography–mass spectrometry (Hogg *et al.*, 2009). Although many samples did not have detectable hormones and concentrations were not quantified, hormone detectability in humpbacks correlated with the sex of the animal (Hogg *et al.*, 2009). Cortisol has recently been reported in humpback blow as well, using ultra-high-performance liquid chromatography (Dunstan *et al.*, 2012). More recently, immunoassay of blow samples from NARWs and belugas has detected a wide variety of steroid and thyroid hormones (Hunt *et al.*, 2012; Thompson *et al.*, 2013). Overall, these studies indicate that the potential is high to use blow samples for endocrine studies, although sampling methodology needs to be tested and standardized, and physiological validations remain to be done.

### Blow microbiology

Blow sampling may provide a unique window into the microbiology and, potentially, the immune status of large whales. The mammalian respiratory tract typically has a diverse microflora, and because it represents a site of potential invasion by pathogens, it is under constant surveillance by the immune system using both active innate and adaptive host responses (Hooper *et al.*, 2012). Therefore, both the microflora and the host immune response can potentially be investigated by analysis of exhaled breath samples.

Efforts to characterize the respiratory microbiome of large whales have recently begun, although studies are in the early stages. In southern resident killer whales, 56 different microbial genera, many of them antibiotic resistant, were isolated from blow droplet samples (Schroeder *et al.*, 2009). Two mysticete species have also been studied recently using pole-based or remote-controlled helicopter sampling methods; β-haemolytic *Streptococcus* spp., *Haemophilus* spp., and *Staphylococcus aureus* were detected in blow of humpback whales (Acevedo-Whitehouse *et al.*, 2010), and *Staphylococcus*, *Pseudomonas*, *Bacillus*, *Streptococcus*, and *Candida* spp. were isolated from blow of western North Pacific grey whales (Denisenko *et al.*, 2012). It is unclear whether these microbial species compose part of the normal respiratory microflora of these species. Further studies involving more individuals of known health status will be needed to characterize the respiratory microbiome profiles of each species fully, to characterize normal vs. pathogenic microbial communites, and to extend the technique to other species. Individual composition variability is apparent; killer whale data suggest that the microbial community varies between individual cetaceans and geographical regions (Schroeder *et al.*, 2009).

### Blow proteomics as a measure of host immune response

Based on studies in other mammals, it is likely that cetacean blow contains a variety of inflammation- and immunity-related compounds, such as fibronectin, lysozymes, immunoglobulin G, cathelicidins, collectins, lactoferrins, and defensins (Brogden *et al.*, 2003; Mitchell *et al.*, 2008). In other vertebrates (mammals and birds), these compounds are involved with wound repair, inflammatory recruitment to sites of infection, and other immune responses (Ganz and Lehrer, 1998; Sugiarto and Yu, 2004). Expression profiles of these peptides, proteins, and their associated genes may reflect the physiological state of the individual. For example, stressed cattle (*Bos taurus*) exhibit alterations in expression of a variety of stress-response proteins (SRPs) in epithelial lining fluid (Mitchell *et al.*, 2008). Although these compounds have not yet been investigated in cetacean blow, parallel studies of cetacean skin (see biopsy sample section) suggest that proteomic techniques developed for terrestrial mammals may be transferable to cetaceans, as long as markers are chosen appropriately (e.g. evolutionarily conserved markers).

### Blow transcriptomics and gene expression

Transcriptomics is the study of all the RNA produced by a cell, tissue, or entire organism at any given time, reflecting the genes that are being expressed at that point in time. Transcriptomic techniques have been applied successfully to cetacean skin (see biopsy sample section) and may also be applicable to blow. Mitochondrial and microsatellite DNA have been isolated from cetacean blow from small odontocetes (Frere *et al.*, 2010), and the potential exists to develop transcriptome techniques as well. This could be a valuable adjunct for assessment of respiratory and systemic immune function and status, e.g. gene expression in cells recovered from exhaled air of cetaceans. For example, characterization of peripheral white blood cell transcript profiles, a technique used successfully in another marine mammal (sea otters, *Enhydra lutris*; Miles *et al.*, 2012), may be possible with whale blow.

### Blow samples: advantages, disadvantages, and next steps

Blow sample analysis offers unique potential for expanding conservation physiology research of large whales, due to the ability to obtain non-invasive samples repeatedly from targeted individuals with a relatively good sampling rate, as well as the wide variety of physiologically relevant measures that appear to be present in mammalian exhaled breath (Table 1). However, blow analysis is still in its infancy, and many validation questions remain to be addressed. Sampling methodology will require detailed study, e.g. determination of the best method for droplet collection, studies of possible interference by sampling materials (e.g. various substrates used for droplet collection), further study of possible methods of acquiring gaseous samples, and addressing potential variation in biomarker content of the sample. Continued collaborations between human breath researchers and cetacean conservation biologists will be most useful.

## Biopsy samples (blubber and skin)

Since the early 1990s, dart biopsying has become one of the most common collection methods for obtaining biological tissue samples from free-ranging cetaceans (Lambertsen, 1987; Mathews *et al.*, 1988; Barrett-Lennard *et al.*, 1996; Noren and Mocklin, 2012). These samples have traditionally provided information about diet (lipid composition and stable isotopes), pollutant exposure, and genetics (Noren and Mocklin, 2012). Increasingly, they are also being used to study the physiological states of sampled individuals, by measuring lipophilic hormones (especially steroids) in the blubber and by characterizing gene expression in the epidermis.

### Biopsy sample collection methods for large whales

**Figure 3:**
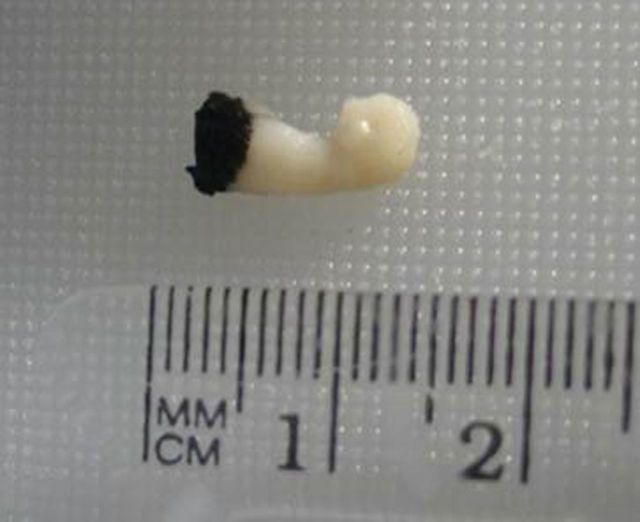
example of a biopsy-dart sample obtained via crossbow, showing epidermis (black tissue) and blubber (white tissue) of a sample obtained from a humpback whale. (Photo: Joanna Kershaw and Christian Ramp, Mingan Island Cetacean Study, Quebec, Canada, permit #QUE02C-2012.)

Dart biopsy samples are predominantly collected using either a crossbow or a pneumatic rifle with modified dart tips (Lambertsen, 1987; Mathews *et al.*, 1988; Barrett-Lennard *et al.*, 1996). The tips are usually hollow, thin-walled, surgical grade stainless-steel cylinders, ∼4–6 mm in diameter and 20–40 mm in length, each with a cutting lead edge and small internal barbs to retract the sample (Barrett-Lennard *et al.*, 1996; Larsen, 1998). The core sample collected from the dart is usually 4–6 mm in diameter with varying lengths, usually between 5 and 30 mm (Fig. 3). This core sample typically consists of both of the two main cetacean skin layers, the outer skin or epidermis (often simply called ‘the skin’) and the dermis (usually called the ‘blubber layer’ or ‘blubber’), consisting largely of adipocytes (Haldiman and Tarpley, 1993; Rommel and Lowenstein, 2001). Here, we will use the terms epidermis and blubber to distinguish these two layers.

It may also be possible to collect epidermal samples in the form of sloughed skin, which is occasionally visible at the water surface after vigorous activites, such as breeching. Such samples have been collected successfully for genetic analyses (e.g. Whitehead *et al.*, 1990; Amos *et al.*, 1992; Valsecchi *et al.*, 1998), but the potential of additional analyses using sloughed skin has not been rigorously explored.

### Blubber hormones

Recently, a nascent but growing field of research has focused on measurement of lipophilic hormones, especially steroids, in blubber (Mansour *et al.*, 2002; Kellar *et al.*, 2006, 2009; Perez *et al.*, 2011). Typically, the tissue has been processed using a multistep organic solvent extraction to isolate lipophilic hormones, and then these hormones are measured using immunoassays or chromatography separation followed by mass spectrometry. Several investigators have used immunoassay of blubber progesterone to assess pregnancy state (in several delphinids, Kellar *et al.*, 2006; minke whale, *Balaenoptera acutorostrata*, Mansour *et al.*, 2002; bottlenose dolphins, *Tursiops truncatus*, and long-finned pilot whales, *Globicephala melas*, Perez *et al.*, 2011). These studies have shown large differences in blubber progesterone of pregnant vs. non-pregnant animals, but no measurable progesterone differences between pregnancy stages. Likewise, in short-beaked common dolphins (*Delphinus delphis*) during the presumed mating period, sexually mature males had substantially higher levels of blubber testosterone than immature males, with no overlap between the two groups (Kellar *et al.*, 2009). Outside the mating period, mature males still had significantly higher testosterone than immature animals, but with more overlap between groups. Thus, blubber hormone analyses may prove to be a useful complement to faecal hormone and blow hormone analyses. Other lipophilic hormones are also likely to be measurable in blubber, including cortisol, aldosterone, and thyroid hormones (Kershaw and Flier, 2004; MacKenzie et al., 2008).

Blubber hormone analysis is still relatively novel. Next steps will involve applying the techniques to more species (particularly mysticetes), and increasing the number of lipophilic hormones validated. As with faecal and blow hormone analyses, validations for large whales will need to include a phase of physiological validations, i.e. determination of normal ranges of various hormones in different demographic groups, using populations that have a high proportion of individuals of known age, sex, and reproductive state. In addition, little is known about the temporal dynamics of hormone deposition in cetacean blubber, i.e. the possible ‘lag’ between hormone increase in blood and subsequent deposition in blubber. Likewise, there may also be lag between reduced levels in blood and clearance from blubber.

### Blubber contaminants

Blubber biopsies have long been used to measure exposure to organic pollutants. Given that cetaceans are long-lived predators, they are predisposed to the accumulation of lipophilic and bioaccumulative contaminants, such as persistent organic pollutants (POPs). Persistent organic pollutants are a significant concern for cetacean health and population sustainability (Tanabe *et al.*, 1994). Most biomonitoring efforts rely on blubber biopsies to assess contaminant exposure in wild cetacean populations, because the blubber is the primary site of accumulation for lipophilic contaminants (Tanabe *et al.*, 1994; Weisbrod *et al.*, 2000; Houde *et al.*, 2005; Balmer *et al.*, 2011; Noren and Mocklin, 2012). Extensive studies have been carried out using blubber biopsy samples to assess the contaminant burdens of a number of cetacean species and populations (Tanabe *et al.*, 1981; Borrell, 1993; Bruhn *et al.*, 1999; Hoekstra *et al.*, 2002; Metcalfe *et al.*, 2004; Tuerk *et al.*, 2005; Krahn *et al.*, 2007, 2008; Litz *et al.*, 2007; McHugh *et al.*, 2007; Pierce *et al.*, 2008; Noël *et al.*, 2009). Overall, males tend to have higher POP concentrations in blubber than females, because females rid themselves of some of their POP burden during lactation. Pollutant levels are positively correlated with increasing trophic level and negatively correlated with body size (Borrell, 1993). Age and sex influences on accumulation must be taken into consideration (Tuerk *et al.*, 2005; Krahn *et al.*, 2009). Generally, analytical procedures for contaminant studies tend to be expensive and time consuming, but the information produced has been invaluable for assessing contaminant exposure and consequent effects on reproduction, mortality, immune function, and other parameters relevant to conservation physiology.

### Blubber lipid analysis

Blubber contains a variety of different fatty acids (FAs), and the types and proportions of FAs present can reveal information relevant to dietary physiology, thermal physiology, and even the age of the individual. Fatty acid profiles have been used to answer qualitative questions about spatial or temporal variation in diets between individuals or populations (Budge *et al.*, 2006), and may be used to some extent to provide a quantitative estimate of diet from FA signatures of predator and prey (quantitative fatty acid signature analysis, QFASA; Iverson *et al.*, 2004). Fatty acid analysis has been used to assess stock structure, trophic positions, diet, and individual age in a variety of mysticetes and odontocetes (Borobia *et al.*, 1995; Dahl *et al.*, 2000; Hooker *et al.*, 2001; Olsen and Grahl-Nielsen, 2003; Herman *et al.*, 2005, 2008, 2009; Thiemann *et al.*, 2007; Walton *et al.*, 2007, 2008; Budge *et al.*, 2008; Loseto *et al.*, 2009; Grahl-Nielsen *et al.*, 2010; Waugh *et al.*, 2012). One complication is that cetacean blubber is highly stratified (Koopman *et al.*, 1996; Krahn *et al.*, 2004; Samuel and Worthy, 2004; Smith and Worthy, 2006), and thus blubber sampling depth can affect FA-based measures. Finally, variations in FA characteristics are known to affect insulative properties of the blubber and thus can be used to study aspects of thermal physiology (pygmy sperm whales, *Kogia breviceps*, and short-finned pilot whales, *Globicephala macrorhynchus*, Bagge *et al.*, 2012).

### Epidermal diseases and microbiology

Epidermal diseases in free-ranging whales and dolphins have been studied primarily using photographic analyses (see next main section), but can also be studied via epidermal and blubber samples. Current understanding of cetacean epidermal diseases is based on samples taken from stranded and bycaught animals (Baker, 1992; Van Bressem *et al.*, 1993; Van Bressem and Waerebeek, 1996; Smolarek Benson *et al.*, 2006), analysed via histology and genetic analysis of samples extracted from lesions. Where possible, collection of biopsy samples from lesions of free-ranging animals would increase our knowledge of pathogens affecting cetaceans and contribute to a better understanding of the pathogenesis of epidermal lesions.

### Epidermal proteomics

A unique aspect of biopsy samples, in comparison to other matrices (faeces and blow), is that biopsy samples contain large quantities of intact peptides and proteins. In terrestrial mammals, proteome-associated profiling is a useful tool to assess energetic balance, immune system function, contaminant exposure, and responses to a diverse range of environmental stressors (Silvestre *et al.*, 2012; Veldhoen *et al.*, 2012), and it is likely that these techniques can be adapted to cetaceans.

A large variety of identification and quantification methods are available for proteomics studies; these include liquid chromatography and two-dimensional polyacrylamide gel electrophoresis (Brewis and Brennan, 2010), tandem mass spectrometry (Dhingra *et al.*, 2005; Brewis and Brennan, 2010), various antibody-based techniques (immunoblotting, immunosorbent assays, competitive binding assays, flow cytometry, etc.; Stoevesandt and Taussig, 2012), gel-free mass spectrometry-based methods, such as differential isotope-coded affinity tags (Stults and Arnott, 2005), and isobaric tagging for relative and absolute quantification (Christoforou and Lilley, 2012)**.** There is, as yet, limited molecular information for many large whales, which limits applications of certain species-specific proteomics tools (Veldhoen *et al.*, 2012). However, those proteins and peptides that appear to be highly conserved across mammals could probably be investigated in large whales with relative ease. Preliminary studies in small odontocetes over the past decade have been encouraging, and indicate that proteomics and transcriptomics may be useful for the types of physiological information outlined below.

### CYP enzymes and contaminant exposure

It has long been known that the mammalian liver expresses a class of cytochrome P450 (CYP450)-dependent monoxygenases or mixed function oxidases after exposure to various hydrocarbons, xenobiotic chemicals, and pharmaceutical drugs (Liska, 1998; Sanchez *et al.*, 2009). Post-mortem liver analyses of sperm whales (Boon *et al.*, 2001) and belugas (*Delphinapterus leucas*; White *et al.*, 1994, 2000; McKinney *et al.*, 2004) have shown that CYP-related enzymes in liver samples are effective biomarkers of contaminant exposure. It is now known that these and related enzymes also occur in the endothelium of the dermal papillae (Bickers *et al.*, 1984; Van Eijl *et al.*, 2012). These enzymes have now been measured in epidermal samples from over 17 species of cetaceans, including both odontocetes and mysticetes (Fossi *et al.*, 1992; Angell *et al.*, 2004), and appear to be useful indices of contaminant exposure (Fossi *et al.*, 1997, 1999, 2008, 2010; Marsili *et al.*, 1998; Wilson *et al.*, 2007; Montie *et al.*, 2008; Waugh *et al.*, 2011).

### Stress-response proteins

Exposure to various stressors often causes systemic cellular stress, characterized by physical and chemical disturbances in the cellular microenvironment, including ionic, pH, and redox changes (Kültz, 2004). These changes, in turn, induce counteracting molecular stress responses, including expression of SRPs that are detectable in many tissues and organs, including the epidermis (Arck *et al.*, 2000). The analytical approach involves assessment of molecular profiles in the epidermis that are indicative of a stress response. For example, spotted dolphins (*Stenella attenuata*) subjected to acute stress associated with chase and purse-seine encirclement exhibited altered SRP expression in epidermal samples, in comparison to control dolphins (Dizon *et al.*, 2002; Southern *et al.*, 2002). The spotted dolphin studies indicate that this method may be suitable for assessing chronic stress (e.g. rather than the acute stress associated with sampling). Additionally, the SRP analytical technique is rapid and relatively inexpensive. The SRPs used in these dolphin studies are thought to be highly conserved across mammals and are likely to be present in large whales as well. Many validations still remain to be done, including age, sex, and stressor relationships in different species, duration of the signal, possible decreased responsiveness to frequently repeated stressors (Southern *et al.*, 2002; Kahan *et al.*, 2009), effect of anatomical sampling location (e.g. jaw, back, and dorsal fin; Dizon *et al.*, 2002), and the relationships between perturbed SRP profiles and long-term health (Dizon *et al.*, 2002).

### Epidermal transcriptomics and gene expression

Transcriptomics has become a widely used approach for the study of normal and diseased gene expression in human skin (Cole *et al.*, 2001; Quan *et al.*, 2009). Some transcriptome studies on cetacean epidermis have been conducted *in vitro* (Ellis *et al.*, 2009; Mollenhauer *et al.*, 2009), and certain genes have been identified that may be particularly useful for cetacean health assessment relevant to conservation issues (Spinsanti *et al.*, 2006). Methods have also been published for sample collection and the development of cetacean cell lines (Marsili *et al.*, 2000), with most studies focusing largely on assessing health, immune system function, or exposure to environmental contaminants. For example, Romano and Warr (2004) have developed genomics-based diagnostic methods for health assessment of bottlenose dolphins (*Tursiops truncatus*) using microarray techniques based on blood-based biomarkers related to immune response and stress. However, application of these microarrays to biopsy samples awaits an assessment of the degree to which these genes are also expressed in dermis and epidermis. Another avenue of research has concentrated on the genes involved in the vitamin D_3_ pathway, which are detectable in dolphin epidermis and may provide information on immune system function (Ellis *et al.*, 2009). Various *in vivo* and *in vitro* studies of dolphins have also investigated altered gene expression profiles in response to contaminant exposure (Mollenhauer *et al.*, 2009; Panti *et al.*, 2011), although long-term implications for health will require further investigation (Panti *et al.*, 2011). Finally, thyroid hormones, thyroid hormone receptors, and certain vitamin A-related measures have also been shown to be disrupted by various environmental contaminants; these measures are detectable in pinniped epidermis and have been shown to reflect contaminant exposure (Tabuchi *et al.*, 2006; Mos *et al.*, 2007), but have not yet been investigated in whales.

### Biopsy samples: advantages, disadvantages, and next steps

In summary, biopsy samples contain a wide variety of invaluable physiological information, ranging from lipophilic hormones and contaminants to CYP-related enzymes, stress-related proteins, RNA and associated indices of gene expression, and potential information on disease states. Use of cetacean biopsy samples in such analyses has two major advantages (Table 1). First, for the last 20 years, biopsies have been routinely collected, resulting in archived collections of tens of thousands of cetacean samples (e.g. The Southwest Fisheries Science Center's Marine Mammal Research Sample Collection, La Jolla, CA, USA). Second, sampling rate is high—to date, higher than for faeces or blow collection—especially when biopsying effort is coupled with traditional line-transect surveying. Up to 75 samples have been acquired from a single species during 1 day of effort from one vessel (Chivers *et al.*, 2010). In fact, several previous studies have resulted in over 1000 samples collected during a single survey season (e.g. Smith *et al.*, 1999). One disadvantage of dart biopsying is that it is invasive (e.g. the skin is penetrated and a small wound created); as a result, certain demographic classes are usually unavailable for sampling due to permit restrictions (Kellar *et al.*, 2013). Fortunately, the small wounds generated by biopsy darting appear to be minor (Noren and Mocklin, 2012), and behavioural responses are minimal (Gauthier and Sears, 1999; Clapham and Mattila, 2006).

In skin sample analyses, an additional challenge for ‘omics’ approaches is divergence in molecular structure and function between model species and marine mammals, particularly regarding comparisons with existing protein and peptide sequence databases. Genome sequences for a few marine mammal species are beginning to emerge, but coverage is still limited. One solution has been to use sequence databases from phylogenetically similar species, thereby allowing identification of highly conserved proteins. As discussed above, there has been parallel interest for developing similar techniques (proteomics, transcriptomics, and genomics) for blow and faecal samples. Overall, the time is ripe to bring the ‘omics’ to the large whales. Such new and emerging molecular approaches will benefit from interdisciplinary research collaborations involving both cetacean field biologists and researchers from other disciplines.

### External appearance of the animal

Visual assessment of the external appearance of an animal is a time-honoured technique for assessing the health and nutritional status of individuals. Subjective assessment has recently been augmented by a variety of semi-quantitative and quantitative methods, which allow more rigorous assessment of changes in large whale body condition, within and between years, in the context of varying food supplies and other conservation-related stressors. The basic premise is that blubber, muscle, and visceral mass increase with nutritional status, enhancing the ability of females to conceive, suckle, and wean offspring, and also enhancing reproductive success of males, and that these changes in mass can be detected by assessment of overall body shape. Furthermore, particular visible aspects, such as skin condition, parasite load and distribution, fresh wounds, and old scars (from vessel and fishing gear interactions) can contribute additional information on health. In small marine mammals, such health assessment can be undertaken by capture and release (Wells *et al.*, 2004), but for the large whales this is impractical, so a series of photography-based studies have been undertaken using remote observational methods.

### Imaging techniques for large whales

**Figure 4:**
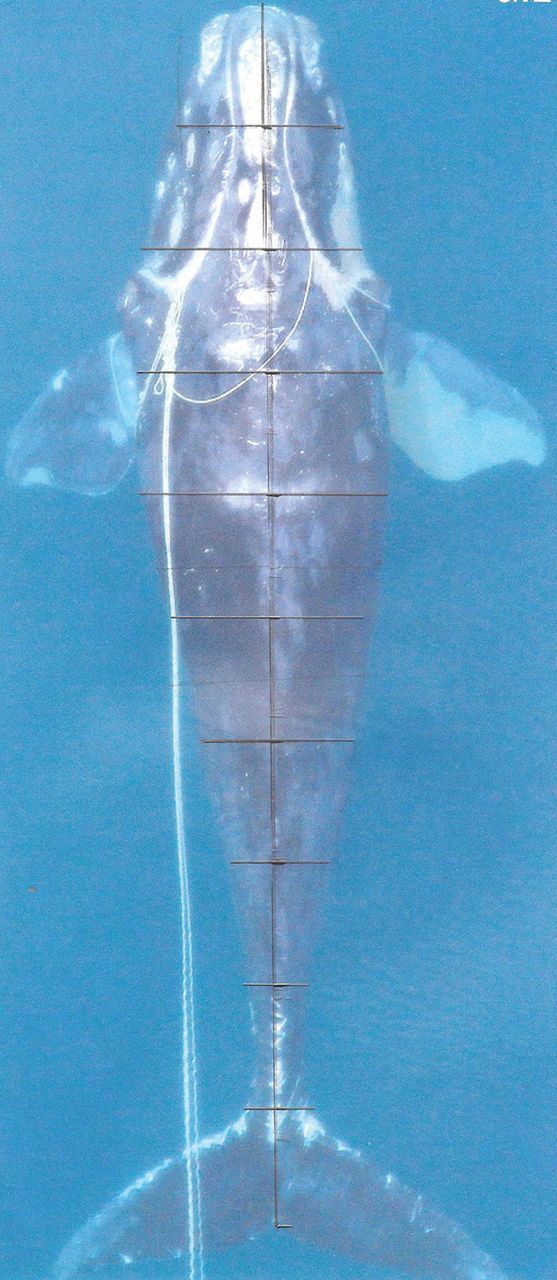
example of aerial photography. This image of an entangled, emaciated North Atlantic right whale (*Eubalaena glacialis*; Eg 3311) has been marked for length-to-width ratio analysis for comparison to unentangled animals, to assess likely body weight prior to dosing with sedatives for disentanglement efforts (van der Hoop *et al.*, 2013). (Photo: Florida Fish and Wildlife Conservation Commission/NOAA, NOAA Fisheries Permit #594-1759.)

**Figure 5:**
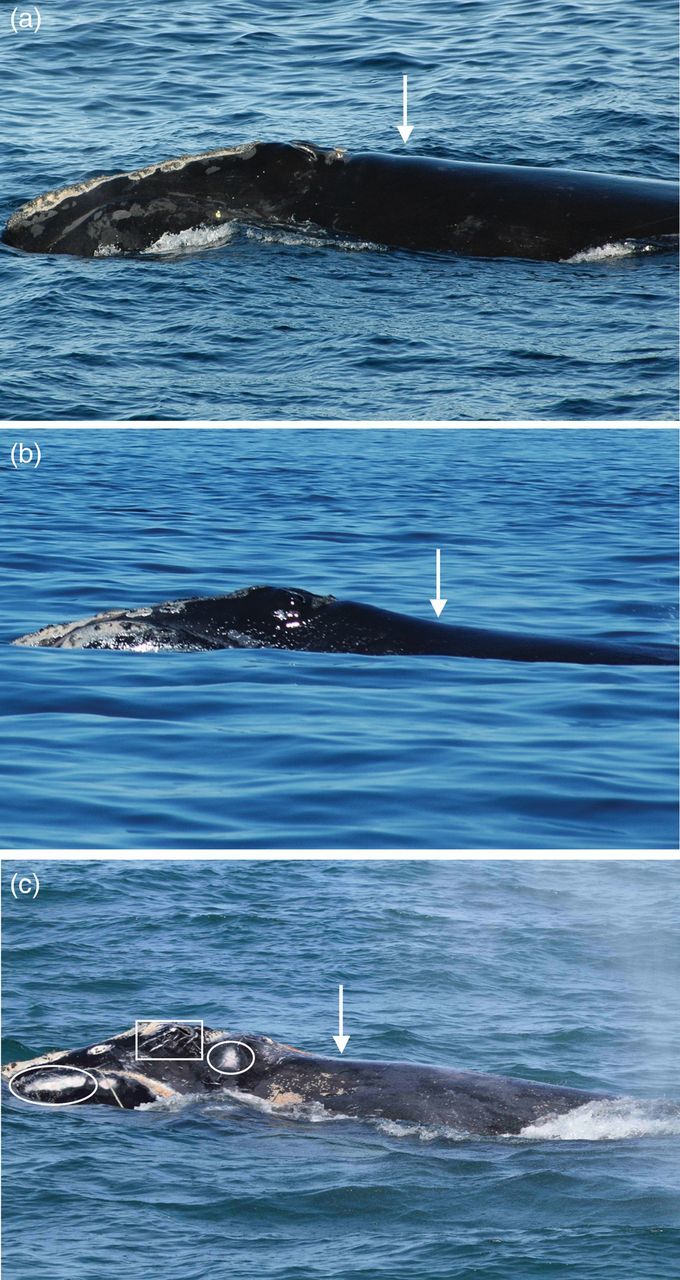
example of boat-based photography. These visual health-assessment photographs show three North Atlantic right whales (*Eubalaena glacialis*) in good (a), fair (b) and poor body condition (c). Body condition is evaluated based on the degree of convexity (good) or concavity (fair or poor) in the dorsal back profile in the post-blowhole area (white arrows). The whale in poor body condition (c) is entangled in fishing line and has other indicators of severely compromised health, including white skin lesions (ellipses) and rake marks forward of the blowholes (rectangle). (Photos a and b: New England Aquarium, SARA permit #322835; Photo c: Georgia Department of Natural Resources, NMFS permit #932-1905/MA-009526.)

Most imaging techniques to assess appearance have been based on photographs from planes (Fig. 4) or boats (Fig. 5). Boat-based photography produces close-up lateral views of whatever portion of the animal is above the surface of the water (typically, the dorsal surface of the animal, and often the entire tail when the animal dives). Aerial photographs are necessarily taken from a greater distance, but if obtained from a perpendicular vantage point above the animal, they provide a unique whole-body perspective that enables measurement of body length-to-width ratios, and true calibrated measurements if altitude and scale are available. Finally, infrared thermography can potentially provide additional information relevant to thermal physiology.

### Aerial photography

Initial aerial measurement of whales at sea primarily focused on measuring body length (Best and Ruther, 1992; Angliss *et al.*, 1995). Body length is often used as a proxy for age in cetacean biology, and many age-related aspects of whale physiology are traditionally expressed in terms of body length (e.g. age of first reproduction). More recently, measurements of length in association with widths in NARWs have allowed estimates of body volume in addition to length (Miller *et al.*, 2012; van der Hoop *et al.*, 2013; Fig. 4), given that necropsy data suggest that widths are predictive of volume and hence mass. In that study, principal components analyses indicated that body width is most variable at 60% of the body length from the snout. Thoracic, abdominal, and caudal body width of southern right whales thinned significantly during the initial months of lactation, especially at 60% of body length from the snout, while their calves' widths and width-to-length ratios increased. Loss of body mass was also observed during lactation. Thus, body width provides a useful measure of nutritional status (Miller *et al.*, 2012).

An integration of these body condition measures has been obtained by quantifying transitions between swimming and gliding for tagged diving whales. Miller *et al.* (2004) showed that sperm whales glide more when buoyancy aids progression. On the ascent, gliding begins once lung expansion overcomes the negative buoyancy of the rest of the animal. Thus, thinner whales, with less fat, will start to glide later during the ascent than fatter animals. Given that body fat can affect diving characteristics, with associated metabolic costs, measures of body condition are highly relevant to many energetic and physiological studies.

### Boat-based photography

Lateral body condition assessment from a boat is only semi-quantitative, given the inability to observe the full submerged body outline, but data indicate that viewing even a portion of the animal may provide useful information on individual health. For example, boat-based photography allows a close-up view of skin condition with a level of detail that is not visible from manned aeroplanes (Hamilton and Marx, 2005). Pettis *et al.* (2004) developed a semi-quantitative visual health-assessment method for NARWs that uses photographs of the visible portions of the head and body (Fig. 5). This method uses a scoring system to evaluate body condition using the following parameters: dorsal blubber profile immediately posterior to the blowholes; skin condition based on the presence, severity, and extent of skin lesions and sloughing; presence and extent of visible ectoparasites (orange cyamids) around the blowholes; and presence of ‘rake marks’ (parallel lines that appear forward of the blowholes of thin animals). Comparison of body condition scores of females during calving and non-calving years indicated that females had poorer body condition in calving years, and also in the year after calving compared with the year before calving. Animals that later disappeared (e.g. presumed dead) had poorer scores than animals that were later resighted alive. Comparison of these body condition scores to blubber thicknesses measured acoustically at sea (Moore *et al.*, 2001; Miller *et al.*, 2011) showed that the semi-quantitative score was less sensitive to subtle differences in body condition detected by blubber thickness measurement, but was very useful for detecting major loss of condition in emaciated animals (Angell, 2005). An extension of this method used on western Pacific grey whales (Bradford *et al.*, 2012) semi-quantified body condition in the post-cranial, scapular, and lateral flank areas, and found that body condition improved significantly as the summer progressed, although not all whales replenished their energy stores by the end of the season. Body condition varied annually, with years of significantly better and worse values. The body condition of lactating females was significantly worse than that of other whales at all times and was most often determined to be compromised.

Photographs can also reveal information on characteristic scars from previous entanglements in fishing gear, enabling analysis of entanglement rate, likelihood of entanglement in different age/sex classes, rate of wound repair and resolution, and associated mortality risks, sublethal costs, and potential short- and long-term physiological effects. Knowlton *et al.* (2012) analysed 30 years of entanglement data on NARWs showing that, on average, 25.9% of adequately photographed NARWs acquired new wounds or scars from fishing gear annually, and 83% of the population had been entangled at least once. Although there was no significant trend in scars over time, the annual percentage of animals observed with rope still on the body (i.e. actual rope, not a scar) increased significantly during the study period, suggesting that it is becoming more difficult for whales to free themselves completely from fishing gear. Parallel studies on entanglement scars in humpback whales suggest that entanglement is a major conservation issue in other species as well; the majority of humpback whales in the Gulf of Maine and also in northern Southeast Alaska suffer an entanglement at some point in their lives (Neilson *et al.*, 2007; Robbins, 2011). Entanglement has been traditionally viewed as an issue of immediate mortality, but an episode of entanglement may also induce long-term physiological costs even if the whale frees itself from the fishing gear, including reduced reproduction, reduced feeding, increased drag with swimming, and increased susceptibility to disease (Knowlton and Kraus, 2001; Kot *et al.*, 2009).

### Infrared thermography

In terrestrial mammals, infrared (IR) photography or ‘thermography’ is increasingly employed for non-invasive studies of thermal physiology (McCafferty, 2007). For cetaceans, IR thermography can only assess the temperature of parts of the animal visible above the water surface, yet even this small portion of the animal can reveal interesting patterns in physiological responses to environmental changes and human stressors. In dolphins, thermal imaging of the dorsal fin has been used to study effects of human disturbance (e.g. prolonged chases, Pabst *et al.*, 2002), as well as seasonal cycles and physiological responses to changes in water temperature (Barbieri *et al.*, 2010). Infrared studies in large whales date back to 1992, when Cuyler *et al.* (1992) used IR thermography to study relationships of body trunk temperatures with ambient water temperatures in five species of baleen whales. Infrared studies of large whales since then have primarily focused on detection of whale presence (e.g. for collision avoidance at night), but lighter and more portable IR imaging systems have become available that may enable greater application of this technique for boat-based physiology studies of large whales (McCafferty, 2007).

### External appearance: advantages, disadvantages, and next steps

Overall, quantitative scoring of visual appearance has proved useful for monitoring nutritional status and general health. Visual appearance in several species has been shown to correlate directly to both reproduction and presumed mortality. Compared with other methods discussed above, visual analysis is entirely non-invasive (like blow and faeces), and also has the great advantage of having by far the highest sampling rate (Table 1). Challenges include the following: relating observations of abnormal skin colouration and texture to specific pathogens or other clinical conditions; revisiting individually recognized animals sufficiently often in the wild to enable meaningful temporal series; and understanding the underlying factors that drive changes in body condition and appearance, such as interannual variation in food availability, human stressors such as background and episodic noise, fishing gear entanglement, and contaminant burden. Aerial imaging provides a desirable whole-body perspective, but has the substantial drawbacks of cost and safety issues. However, it may be possible to use remote-controlled hexacopters as a low-cost option for aerial photography that could be operated from small boats at sea (W. Perryman, personal communication, NOAA SW Fisheries Science Center, La Jolla, CA, USA). Another limitation of the aerial method is the difficulty of accurately assessing the maximal width at each point on the animal when it may be submerged in water that is variably opaque.

Next steps should amalgamate assessment of body condition in photo-identified whale populations with a better understanding of cumulative human impacts upon their rates of morbidity and mortality. For example, long-term photographic assessment of entanglement rates (e.g. Knowlton *et al.*, 2012) could be combined with other measures, such as aerial estimates of nutritional status (Miller *et al.*, 2012), close-up analyses of skin appearance and ectoparasite load (Pettis *et al.*, 2004), and assessment of subsequent impacts on reproduction (both via calving rate and via endocrine approaches discussed above). Such multi-analytical approaches would be very informative about the sublethal costs of human impacts.

## Summary and conclusions

In contrast to the dearth of physiological information available from large whales in the past, we now have three types of physiological samples that are fairly readily obtainable from whales (faeces, blow, and biopsy samples) as well as an increasing amount of physiological data that can be gleaned from visual assessment. All four methods are feasible to implement from a field perspective. Many, if not most, cetacean research teams already routinely take photographs and collect biopsy samples during population surveys; many teams also have considerable skill with using cantilevered poles around whales (for disentanglement, tagging, etc.) and can readily adjust this methodology to blow sampling; and faecal samples are easily scooped up opportunistically (although sampling rate is typically higher with a dedicated boat, and higher still with a trained dog). Endocrine studies can potentially take advantage of three possible sample types—faeces, blow, and blubber—with all three matrices being likely to represent different time frames for acute vs. chronic endocrine changes. Furthermore, each sample type can provide separate information on specific organ systems, e.g. blow for respiratory physiology, faeces for digestive physiology, biopsies and photographs for the integument, and biopsies for fat stores. Finally, the possibility of developing a remote blood-sampling apparatus should not be discounted; creative engineering approaches might yet devise a solution to this difficult problem.

Whatever the sample type, the value of beginning with well-studied populations of known individuals cannot be overstated. For all the techniques discussed here, assay validation and careful development using populations with known individuals has been extremely useful. Specific examples include the well-studied NARW population in the Bay of Fundy, the southern resident killer whale population of Puget Sound, and certain populations of humpbacks. We encourage researchers to take advantage of well-known, photo-identified populations when translating novel analytical methods from terrestrial species and small captive odontocetes to large free-swimming whales. When feasible, testing and validating methods with display or rehabilitated animals would also be invaluable. Once these techniques have been validated and proven in several baleen species and several odontocetes, we consider it likely that the techniques can then be applied to other populations that may not have known individuals, especially if study designs take advantage of comparison of populations before vs. after an event, or comparisons across populations exposed to different conservation pressures.

We expect that combinations of different techniques will provide useful cross-checks and cross-validations. For example, stress assessment, a common issue in conservation management, can be approached via multiple independent techniques, potentially including the following: changes in faecal, blow, and blubber glucocorticoids (bearing in mind the possibility of depression below normal, e.g. ‘adrenal insufficiency’, in cases of severe long-term stress); possible depression of thyroid hormones in cases of long-term chronic stress (particularly long-term nutritional stress); depressed reproductive hormones in long-term chronic stress (e.g. decreased androgens of adult males, and decreased progesterone and oestrogens in females); loss of body condition (as assessed from photographs); potentially decreased immune measures and increased SRPs; and so forth. Correspondence of such patterns across matrices and across techniques, and subsequent comparison to impacts on reproduction and mortality, would provide powerful evidence that the methods used can provide valid ‘early warning’ information indicating significant conservation impacts.

The study of large whale physiology is now at a stage at which a ‘critical mass’ of non-lethal assessment methods has become available. Together, faecal samples, blow samples, biopsy samples, and visual assessment methods have the potential to revolutionize our understanding of large whale reproductive cycles, stress physiology, nutritional status, host immune response, pathogen and parasite load, and more. Ultimately, these data can be used to assess how these physiological parameters are affected by the numerous conservation pressures impacting many large whale populations today. Further development of these techniques could identify which measures will be most useful as early warning indicators of potentially serious sublethal (or even lethal) impacts and which may indicate long-term chronic impacts. As a next step, conservation physiology studies of large whales will benefit greatly from cross-disciplinary approaches that include cetacean conservation researchers along with experts from other fields (e.g. endocrinology, proteomics, genomics, microbiology, and human breath research), along with further testing on well-studied populations of large whales.
